# A Whole-Genome-Based Gene-by-Gene Typing System for Standardized High-Resolution Strain Typing of Bacillus anthracis

**DOI:** 10.1128/JCM.02889-20

**Published:** 2021-06-18

**Authors:** Mostafa Y. Abdel-Glil, Alexandra Chiaverini, Giuliano Garofolo, Antonio Fasanella, Antonio Parisi, Dag Harmsen, Keith A. Jolley, Mandy C. Elschner, Herbert Tomaso, Jörg Linde, Domenico Galante

**Affiliations:** a Institute for Bacterial Infections and Zoonoses, Friedrich-Loeffler-Institut, Jena, Germany; b Department of Pathology, Faculty of Veterinary Medicine, Zagazig University, Zagazig, Sharkia Province, Egypt; c Istituto Zooprofilattico Sperimentale dell’Abruzzo e del Molise G. Caporale, Teramo, Italy; d Anthrax Reference Institute of Italy, Istituto Zooprofilattico Sperimentale della Puglia e Basilicata, Foggia, Italy; e Department of Periodontology and Operative Dentistry, University Hospital Muenster, Muenster, Germany; f Department of Zoology, University of Oxford, Oxford, United Kingdom; National Institute of Allergy and Infectious Diseases

**Keywords:** *Bacillus anthracis*, genome typing, cgMLST, canonical SNP, whole-genome typing

## Abstract

Whole-genome sequencing (WGS) has been established for bacterial subtyping and is regularly used to study pathogen transmission, to investigate outbreaks, and to perform routine surveillance. Core-genome multilocus sequence typing (cgMLST) is a bacterial subtyping method that uses WGS data to provide a high-resolution strain characterization. This study aimed at developing a novel cgMLST scheme for Bacillus anthracis, a notorious pathogen that causes anthrax in livestock and humans worldwide. The scheme comprises 3,803 genes that were conserved in 57 B. anthracis genomes spanning the whole phylogeny. The scheme has been evaluated and applied to 584 genomes from 50 countries. On average, 99.5% of the cgMLST targets were detected. The cgMLST results confirmed the classical canonical single-nucleotide-polymorphism (SNP) grouping of B. anthracis into major clades and subclades. Genetic distances calculated based on cgMLST were comparable to distances from whole-genome-based SNP analysis with similar phylogenetic topology and comparable discriminatory power. Additionally, the application of the cgMLST scheme to anthrax outbreaks from Germany and Italy led to a definition of a cutoff threshold of five allele differences to trace epidemiologically linked strains for cluster typing and transmission analysis. Finally, the association of two clusters of B. anthracis with human cases of injectional anthrax in four European countries was confirmed using cgMLST. In summary, this study presents a novel cgMLST scheme that provides high-resolution strain genotyping for B. anthracis. This scheme can be used in parallel with SNP typing methods to facilitate rapid and harmonized interlaboratory comparisons, essential for global surveillance and outbreak analysis. The scheme is publicly available for application by users, including those with little bioinformatics knowledge.

## INTRODUCTION

Bacillus anthracis is a Gram-positive, rod-shaped, spore-forming bacterium and the etiological agent of anthrax in wildlife, livestock, and humans worldwide (1). This bacterium is notorious as an agent of bioterrorism and as a state-sponsored biological weapon associated with severe infections and outbreaks around the world (2). B. anthracis produces spores that remain viable in the soil and can be dispersed by wind and vectors (1). Livestock grazing in regions of anthrax endemicity can take up anthrax spores, while humans can be directly infected when they handle anthrax-infected animals, eat contaminated food, or inhale anthrax spores (3). Humans develop different forms of anthrax, including cutaneous, gastrointestinal, and inhalational forms, as well as injectional anthrax reported in drug users (4). B. anthracis has two toxin-carrying plasmids (pXO1 and pXO2) that can also be present in other *Bacillus* species leading to atypical anthrax-causing strains (5).

B. anthracis harbors a highly monomorphic genome with a very limited genetic diversity. Strains of B. anthracis are highly clonal, with more than 99.9% average nucleotide identity (ANI) compared to the Ames Ancestor strain (NC_007530.2) (6). Standard genotyping methods for B. anthracis include canonical single-nucleotide polymorphisms (SNPs) and multilocus variable-number tandem repeat (VNTR) analysis (MLVA). Canonical SNP typing employs a selection of representative branch-specific SNPs for phylogenetic branches, which can be used to define key phylogenetic positions for descendant strains (7–9). Based on canonical SNP analysis (8–10), B. anthracis can be divided into three major lineages—A, B, and C—that subdivide into sublineages with a typical geographical distribution (10). MLVA has higher discriminatory power (5) and uses tandem repeats to further subgroup B. anthracis strains via analyzing 8 (11), 15 (9), 20 (12), 25 (13), or 31 (14) VNTR loci. However, MLVA is laborious and prone to homoplasy problems (5). Today, whole-genome sequencing (WGS) is the method of choice to identify genome-wide SNPs in B. anthracis, which allows the definition of new canonical genetic lineages (7, 15), but also greatly enhances the resolution of phylogenetic analyses in outbreak settings. Core-genome-based multilocus sequence typing (cgMLST) is a genome-wide typing system for a high-resolution clustering that indexes strain genotyping results into allelic numbers that can be accessed via a central database. In addition to the established SNP typing, this central curated database for B. anthracis WGS-based typing can be used for routine surveillance and tracing outbreaks back to the source. Several studies have described the successful utilization of cgMLST in outbreak and epidemiological analyses of different pathogens (16). While commercial tools are established (17), international organizations like the European Union support freely available tools (18). In fact, free online tools for cgMLST have been established that may allow usage of cgMLST by non-bioinformaticians (19). Therefore, we aimed to develop and validate a new cgMLST for B. anthracis as a tool for gene-by-gene comparison using WGS data.

## MATERIALS AND METHODS

### Bacillus anthracis strains and genomes.

A total of 753 B. anthracis strains were used for scheme setup, application, and validation (see Table S1 in the supplemental material). First, we selected 57 B. anthracis genomes (referred to as query genomes) available at RefSeq (April 2019) and used for the cgMLST scheme setup.

Then, for scheme application and validation, we used three data sets. Data Set 1 was aimed at (i) investigating the overall cgMLST scheme typeability (defined as the percentage of genes assigned allele numbers per strain) and (ii) investigating the backward compatibility of the new scheme to standard canonical SNP grouping to classify B. anthracis into major clades and subclades as previously defined and accepted by anthrax laboratories around the globe (20). This data set was investigated in previous studies (7, 20–22) and includes sequence data of 596 B. anthracis strains that cover the global population diversity of B. anthracis and were recovered over the last 110 years from 50 countries on six continents (Table S1).

Data Set 2 was used to delineate allelic distances between epidemiologically linked strains. This data set (total = 43) includes (i) eight epidemiologically related strains from the three most recent anthrax outbreaks in Germany in 2009, 2012, and 2014, as well as (ii) 35 epidemiologically unrelated strains from 35 different outbreaks in Italy with one strain per outbreak (Table S1). Strains of this data set were sequenced in this study, except three German strains previously sequenced using the Ion Torrent or Pacific Bioscience platform (23–25). Data Set 3 includes 57 published B. anthracis strains isolated from heroin users in four European countries (26) (Table S1) and was used to evaluate the proposed cutoff for clustering.

### Whole-genome sequencing and *de novo* assembly.

B. anthracis strains (Data Set 2) were sequenced using Illumina MiSeq (Illumina, USA). DNA was extracted using the Genomic-tip 100/G and genomic DNA buffer kit (Qiagen, Germany) for the German strains. For the 35 strains from Italy, the DNAeasy blood and tissue kit (Qiagen, Germany) was used. Paired-end sequencing libraries were prepared using the Nextera XT DNA Library Preparation kit (Illumina, USA) with an average sequencing depth of between 40× and 104× for the strains. Genome assembly was performed for sequence data produced in this study or downloaded from the NCBI using shovill v.1.0.4 (option -trim [https://github.com/tseemann/shovill]) for paired-end Illumina data or SPAdes v.3.12.0 (-careful option) (27) for single-end Illumina data. Assembly statistics were obtained using the program Quast v.5.0.2 (28).

### Development and application of a cgMLST.

For scheme setup, we first investigated the genetic population structure of B. anthracis using canonical SNPs and Bayesian analysis to select representative genomes of all phylogenetic groups. For that, we used 172 genomes from the RefSeq database (Table S1). Briefly, we estimated the pairwise average genomic nucleotide identity between all genomes using pyani v.0.2.9 (module ANIm) (29) and FastANI (30). We then used Parsnp v.1.2 (31) to build a core-genome SNP alignment with the “Ames Ancestor” strain as a reference. The genetic population diversity of the strains was inferred based on hierBAPS grouping (32) using Python script from Bruce et al. (21) and canonical SNPs as inferred from the SNP-based phylogenetic analysis following nomenclature proposed by Sahl et al. (20) and Van Ert et al. (9). Based on their representativeness of global phylogeny of B. anthracis and the completeness of genome assemblies, we selected 57 B. anthracis genomes (referred to as query genomes) (see Fig. S1 and Table S1 in the supplemental material).

The cgMLST Target Definer tool v.1.5 within Ridom SeqSphere+ v.7.1.0 (17) was used to define cgMLST targets. The genome sequence of the Ames Ancestor strain (NC_007530.2) was used as a reference. The following criteria were applied to exclude reference genes that (i) have a length of less than 50 bp (“minimum length filter”), (ii) lack a start and/or a stop codon (“start codon filter” and “stop codon filter”), (iii) have paralogues with identity of >90% and overlap of >100 bp (“homologous gene filter”), and (iv) are overlapping large genes (“gene overlap filter”). Retained genes from the reference strain were then searched through BLAST v.2.2.12 against the 57 query genomes. Genes lacking a single start or stop codon in 80% of the query genomes as well as genes that match plasmid sequences by BLAST analysis (NC_007322.2 and NC_007323.3) were excluded. BLAST thresholds include 90% identity and 100% coverage, with parameters involving a word size of 11, mismatch penalty of 1, match reward of 1, gap open costs of 5, and gap extension costs of 2.

The application of the cgMLST was performed using Ridom SeqSphere+ v.7.1.0 (17). This involves (i) BLAST detection of the cgMLST genes in the genomes with sequence identity of >90% and overlap of >99% and (ii) assigning allele numbers for gene sequences. SeqSphere+ v.7.1.0 assigns allele numbers with the following quality metrics: the gene has no frameshift, has no ambiguities (only AGCT characters), has start and stop codons, and is of a length equal to that of the reference gene ± 3 codons. Since the cgMLST system is sensitive to assembly artifacts and errors, we excluded genomes with an *N*_50_ of less than 40 kb from the evaluation data sets. For all investigated genomes, cgMLST allelic profiles (a combination of alleles number per each strain) were compared pairwise (untypeable genes were ignored), and the resultant calculated pairwise distances were used to generate a neighbor-joining (NJ) tree and a minimum-spanning tree (MST) using SeqSphere+. Genes were regarded untypeable if they were not assigned an allele number because of no BLAST match or due to the incidence of internal stop codons or ambiguities in the gene.

The developed cgMLST scheme was incorporated into the PubMLST Bacillus cereus database (https://pubmlst.org/) together with gene-allele libraries of the B. anthracis genomes (19). In additional to access via the website, the scheme is also accessible via the PubMLST RESTful application programming interface (https://rest.pubmlst.org) (33). We have additionally incorporated characteristic anthrax plasmid genes for pXO1 (*cya*, *lef*, *pagA*, and *repX*) and pXO2 (*capA*, *capB*, *capC*, *capD*, *capE*, and *repS*) in the database as previously described (34). Finally, the scheme was formatted and imported from SeqSphere+ into the chewBBACA software (18), a command line-based open-source program for allele calling that can be implemented in customized standalone pipelines. The snakemake (35) application of chewBBACA, called chewieSnake v.2.0.0-52-g01c32bb (accessed July 2020 [https://gitlab.com/bfr_bioinformatics/chewieSnake]) was used with default options, including 0.6 as the minimum BLAST score ratio for locus similarity (–bsr 0.6) and 20% length boundaries for allele definition (–st 0.2). GrapeTree (36) was used to compute the allele distance matrices, with missing data being ignored in comparisons. The cgMLST profiles identified using chewBBACA and SeqSphere were compared using Simpson's index of diversity and an adjusted Wallace test of congruence using the Comparing Partitions tool (37).

### Whole-genome SNPs.

We used Parsnp v.1.2 (31) to identify the whole-genome SNPs. The Ames Ancestor strain was used as a reference, and all genomes of Data Set 1 were included. Putative recombination sites were removed using Gubbins v.2.2.1 under default settings. Trees constructed based on core-genome SNPs and cgMLST were compared for topology concordance using the tanglegram algorithm in Dendroscope v.3.2.1027 (38).

### Data availability.

Raw sequencing data generated in this study are available under NCBI Bioproject accession no. PRJNA656733. Additional sequence data analyzed in this work are available in the NCBI SRA database and listed in Table S1.

## RESULTS

### Calculation of B. anthracis cgMLST targets.

Of the 5,357 genes with CDS in the chromosome of the Ames Ancestor strain, 511 genes were excluded due to gene overlapping (*n = *220) or repetition (*n = *26) or because the genes had internal stop codons (*n = *265) (17). Additionally, reference genes that were missing (*n = *1,025) in any of the 57 query genomes or carrying an internal stop codon (*n = *2) in >80% of the query genomes were discarded. Following these criteria, we determined 3,803 core genes were suitable for cgMLST typing comprising 58.2% (3,047,205 bp) of the reference genome (see Table S2 in the supplemental material). These genes have an average GC content of 36.02% and average length of 801.3 bp (minimum of 81 bp and maximum of 7,158 bp).

### Evaluation and validation of the cgMLST scheme.

We used a set of 596 publicly available B. anthracis genomes to evaluate the applicability of the cgMLST scheme (see Data Set 1 in Table S1). Twelve genomes (out of 596) did not belong to the highly clonal B. anthracis lineage and had less than 90% of the cgMLST targets. The pairwise ANI values for these strains were 93 to 98% compared to the B. anthracis reference genome (Table S1). For the remaining 584 strains (out of 596) of classical B. anthracis lineage, the 3,803 cgMLST targets were identified on average with 99.1% (standard deviation, 0.63%) per genome, with more than 99% of the cgMLST genes being conserved and typed in a total of 382 genomes (∼65.4% of the data set). Decayed genes (genes with internal stop codons) represented the majority of untypeable genes (mean, 34.7 ± 27.4) (Table S1). The 584 genomes were clustered into 473 cgMLST profiles (missing alleles are ignored in pairwise comparisons), resulting in a Simpson’s index of discrimination of 0.998 (95% confidence interval [CI], 0.998 to 0.999). This was slightly less than the number of unique SNP profiles (*n *= 508) identified for the 584 genomes based on 15,081 whole-genome SNPs, with a Simpson's index of diversity of 0.999 (95% CI, 0.998 to 0.999). Furthermore, there was a high topological concordance between cluster analysis inferred from cgMLST and the maximum likelihood tree based on whole-genome SNPs (see Fig. S2 in the supplemental material). These results emphasize that the developed scheme provides enough discrimination between strains and is highly concordant to whole-genome SNPs.

**FIG 1:**
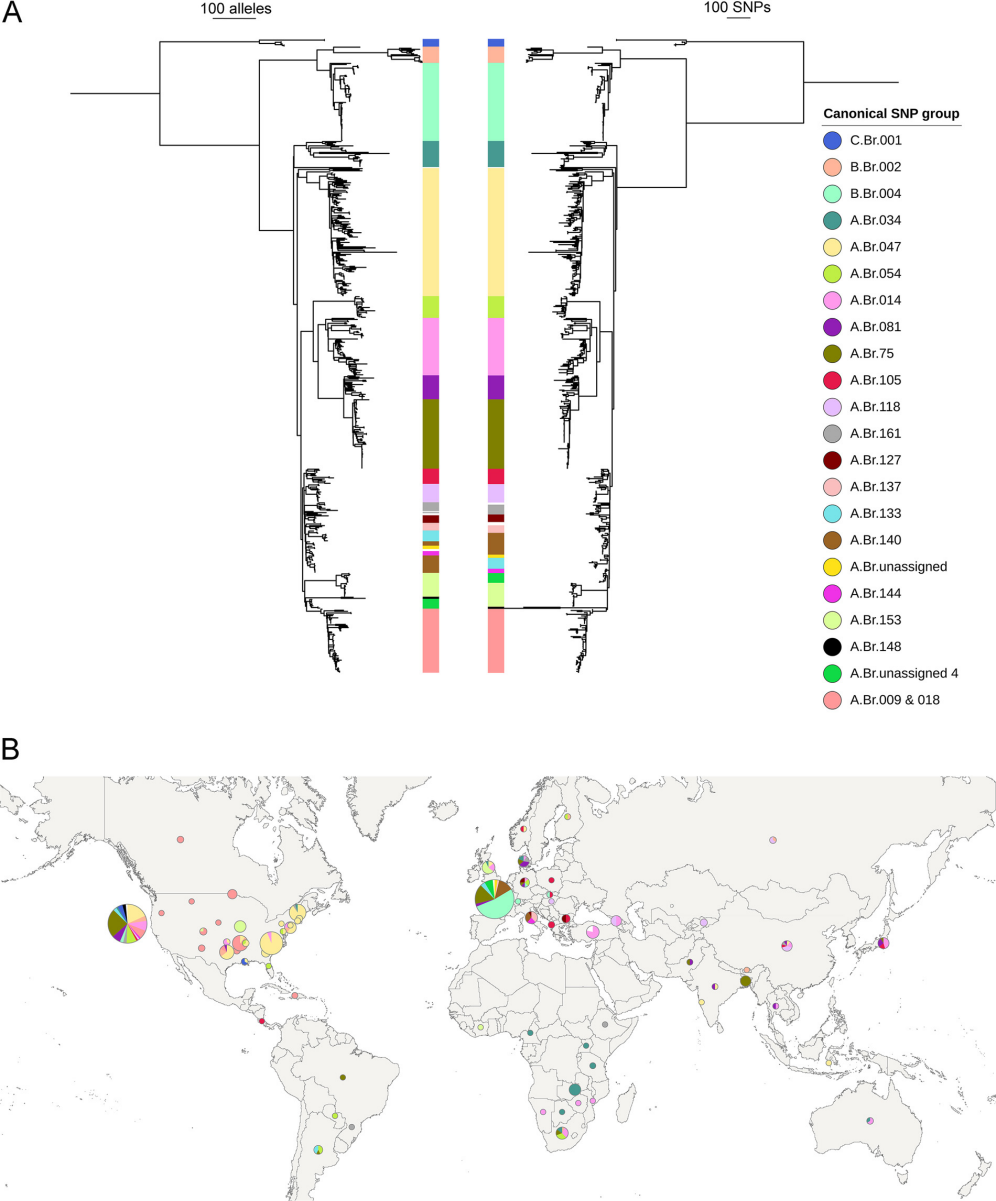
Phylogenetic analysis and geographical origin distribution of global B. anthracis genomes. (A) Comparison between the neighbor-joining tree (left) and maximum likelihood tree (right) constructed for the 584 genomes based on the pairwise allelic distances, ignoring untypeable genes and whole-genome SNPs after filtering regions with high SNP density using Gubbins, respectively. Tree visualizations were performed using iTOL. (B) Geographical origin distribution of 584 B. anthracis genomes used in the evaluation of the core-genome MLST. The updated canonical SNPs groups from Sahl et al. (20) were added and color coded.

As presented in Fig. 1, the inference of the global phylogeny of B. anthracis using the cgMLST profiles clearly defines the three different A, B, and C clades of B. anthracis (20). Clade A comprises 84% of the data set (*n = *481), followed by clade B (*n = *87 [14.8%]), and then clade C (*n = *7 [1.9%]). Clade A splits into six subclades, with subclade “Ancient A” being basal to all other subclades and the subclades “V770,” “Sterne/Ames,” and “Australia 94” having evolved from a single branch (A.Br.004). Only one strain could not be assigned to any of the defined subclades of clade A. The subclade “TransEurAsia” (TEA, or A.Br.008) is the most abundant, comprising ∼32% of the data set (*n = *188), with a very wide geographic distribution in Europe (*n = *88), North America (*n = *72), Asia (*n = *23), South America (*n = *3), and Africa (*n = *2). Although trees based on cgMLST and SNPs resulted in the same subclade definition, there were some differences in the branching points between both trees that led to switches in the positions of a few groups of the TEA subclade.

The average allele distance within and between each of the identified (sub)clades and the average allele distance of each (sub)clade to the closest sample in the other groups show that cgMLST can discriminate between the established subgroups of B. anthracis (see Table S3 in the supplemental material). We also identified specific SNPs within the cgMLST targets that are specifically present in each of the B. anthracis (sub)clades but simultaneously absent in all other genomes (see Table S4 in the supplemental material). These group-specific SNPs are positioned within the identified cgMLST targets and can serve as further targets for fast identification of new strains (Table S4).

**FIG 2:**
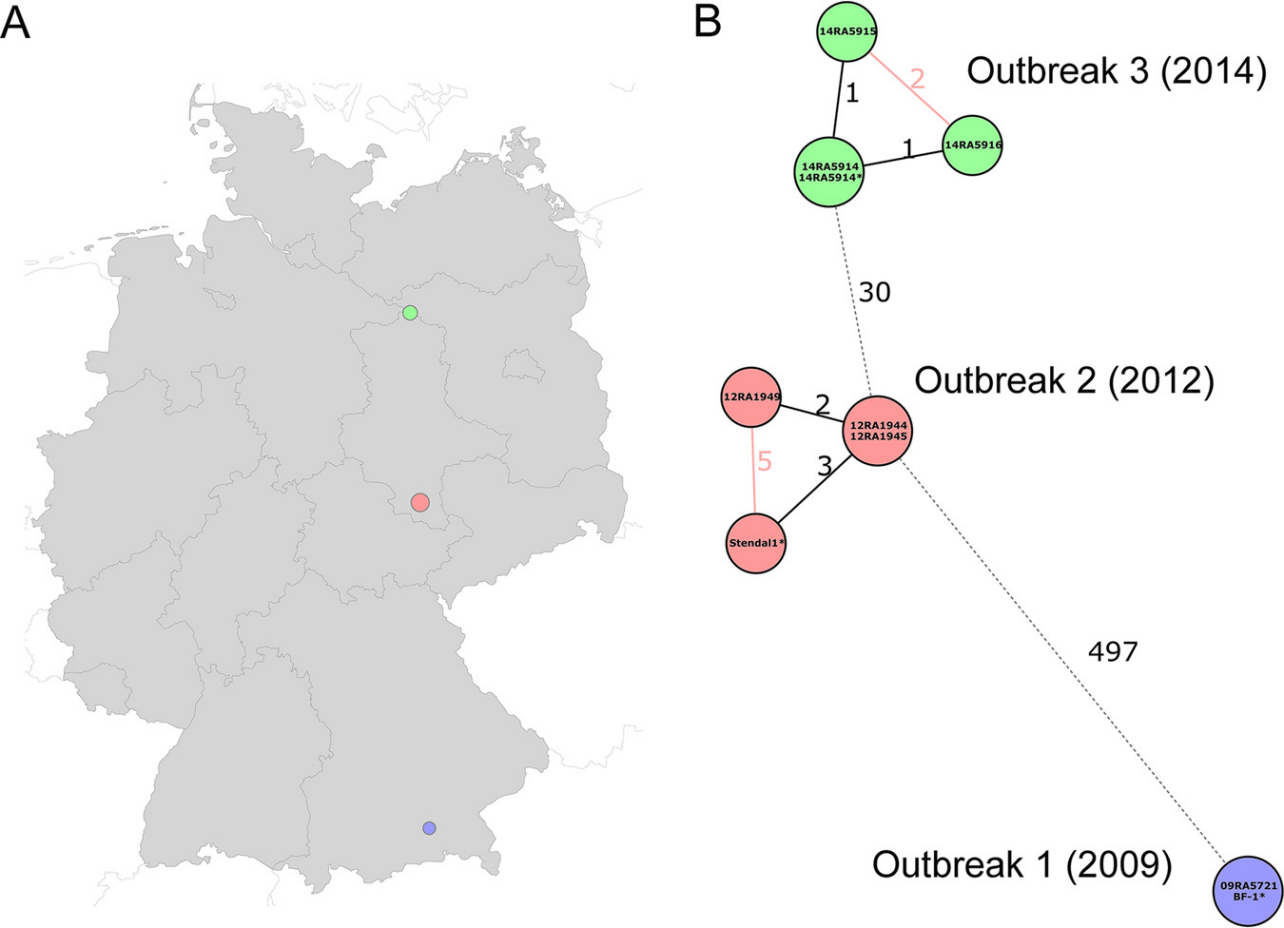
Geographical origin distribution (A) and a minimum-spanning tree (B) illustrating the last three anthrax outbreaks that occurred in cattle populations in Germany. Each node represents a unique cgMLST allele profile. Colored nodes represent the location of isolation. Numbers on connecting lines refer to the number of different alleles. Previously published genomes are marked with a star (*).

In order to delineate outbreak strains in the future, we investigated sequence data of known epidemiologically linked strains (*n *= 8) and strains that have no epidemiological relationships (*n *= 35). For the epidemiologically linked strains, we investigated sequence data of the three most recent anthrax outbreaks that occurred in the cattle population in Germany since 2009 (Fig. 2; Data Set 2 in Table S1). The first anthrax outbreak occurred in 2009 in the Bavarian Alps and caused the deaths of five cows. From this outbreak, strains BF-1 (23) and 09RA5721 were isolated and sequenced in two laboratories. In July 2012, a second anthrax outbreak occurred in the county of Stendal (Saxony-Anhalt), which resulted in 10 losses out of 55 cattle in the affected farm. Three isolates were retrieved: one from the spleen of a cow and the other two from blood culture and lymph node of another cow. Finally, the last anthrax outbreak was reported in 2014 in Dobichau in Saxony-Anhalt and resulted in four dead animals. Three B. anthracis strains were isolated from spleen, kidney, and synovial fluid of a single cow. Classical typing using canonical SNPs and MLVA identified identical B. anthracis strains involved in the two outbreaks in Saxony-Anhalt in 2012 and 2014. The isolates belong to the same canonical SNP group, A.Br.001/002, and share identical MLVA profiles based on extensive analysis of 31 VNTR loci (W. Beyer [http://microbesgenotyping.i2bc.paris-saclay.fr/databases]). The 2009 outbreak was distant from the other outbreaks and caused by a clade B strain. Using cgMLST, a maximum of five allelic variations could be identified between isolates from a single outbreak (Fig. 2). This was observed for the strains recovered from different animals or different organs in the same animal. The cgMLST showed that the two outbreaks in Saxony-Anhalt were due to strains that were distant from each other by 30 different alleles, which might indicate different sources of the outbreaks, precluding a direct transmission of strains between these outbreaks.

**FIG 3:**
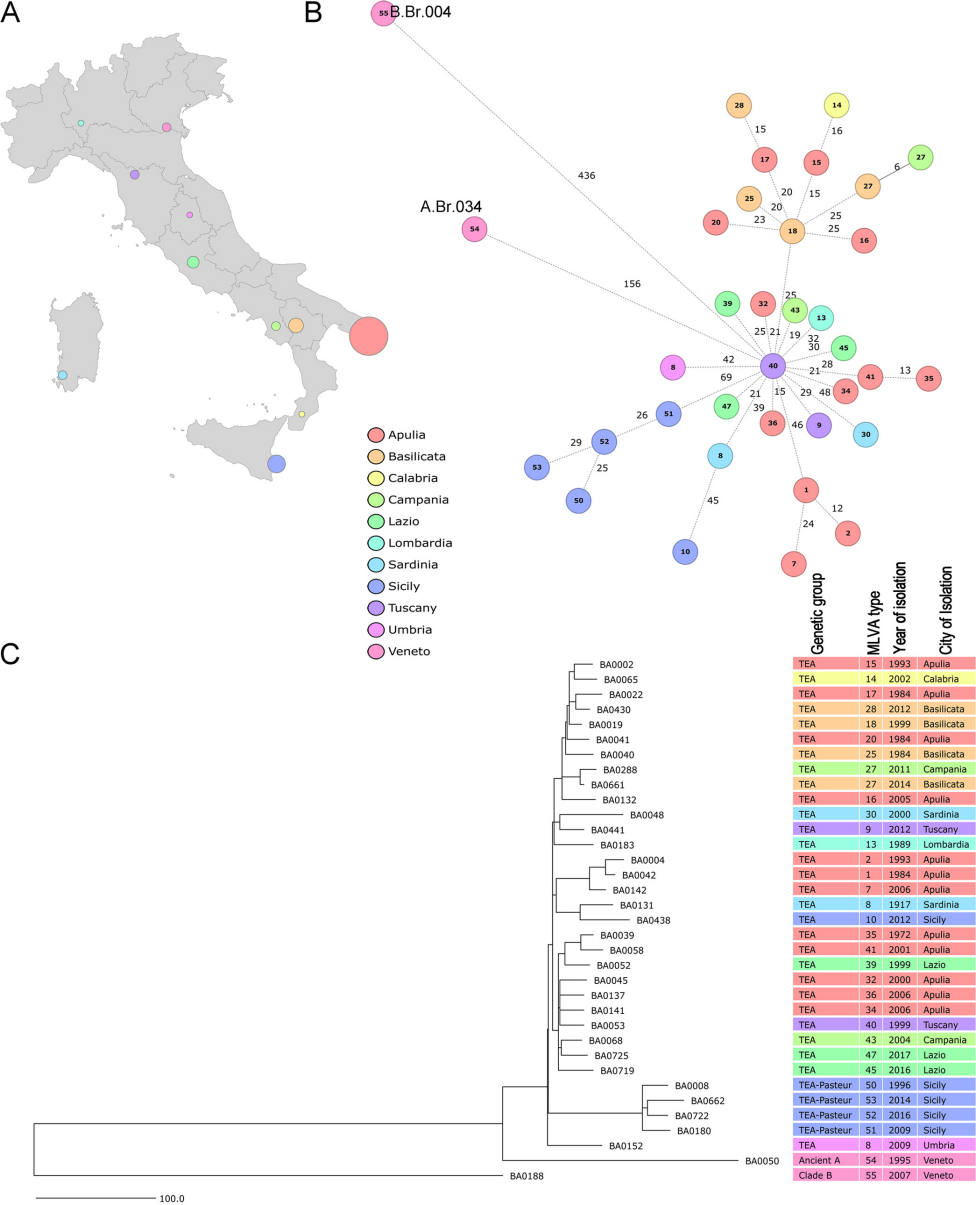
Geographical origin distribution (A) and minimum-spanning tree (B) illustrating 35 distinct spatiotemporal anthrax outbreaks that occurred in Italy. Each node represents a unique cgMLST allele profile. Colored nodes represent the city of isolation, while node labels correspond to different MLVA profiles. Numbers on connecting lines refer to the number of different alleles. (C) Phylogenetic analysis of the strains using a neighbor-joining tree based on the whole-genome SNP data.

We also investigated 35 strains recovered from different hosts and regions in Italy (Fig. 3; Data Set 2 in Table S1). This includes Basilicata (*n = *4), Apulia (*n = *12), Sicily (*n = *5), Sardinia (*n = *2), Tuscany (*n = *2), Veneto (*n = *2), Lazio (*n = *3), Campania (*n = *2), Calabria (*n = *1), Lombardia (*n = *1), and Umbria (*n = *1). The strains were recovered from cattle (*n = *21), sheep (*n = *8), goats (*n = *4), a horse (*n = *1), and a human (*n = *1) (Table S1). The cgMLST analysis confirmed classical canonical SNP typing in which one strain belong to clade B (B.Br.004) and one strain of subclade Ancient A (A.Br.034). The remaining 33 strains belong to the TEA (A.Br.008) group (28 strains of A.Br.011/009 and 5 strains of A.Br.008/011 [Pasteur]). MLVA typing classified 31 strains as singletons. Two MLVA genotypes, MLVA31-8 and MLVA31-27, comprised two strains each. However, MLVA31-8 was found to contain strains that differed in 58 alleles, as determined using cgMLST. MLVA31-27 contained strains that differed by six alleles, as determined using cgMLST (Fig. 3). These results indicate that cgMLST provides better resolution than MLVA and can resolve ambiguities associated with MLVA genotyping.

In this study, we propose a cutoff threshold of five alleles to be used to trace epidemiologically linked strains for cluster typing and transmission analysis in B. anthracis. This is based on our observation that strains recovered from the same outbreak can vary by up to five alleles. This might occur due to microevolutionary changes during an outbreak or during subsequent strain culturing, or it might be due to a sequencing/assembly error that impaired gene composition. The five-allele rule is not a hard and fast proven benchmark, but rather the starting point for understanding and comparing outbreaks in the future.

**FIG 4:**
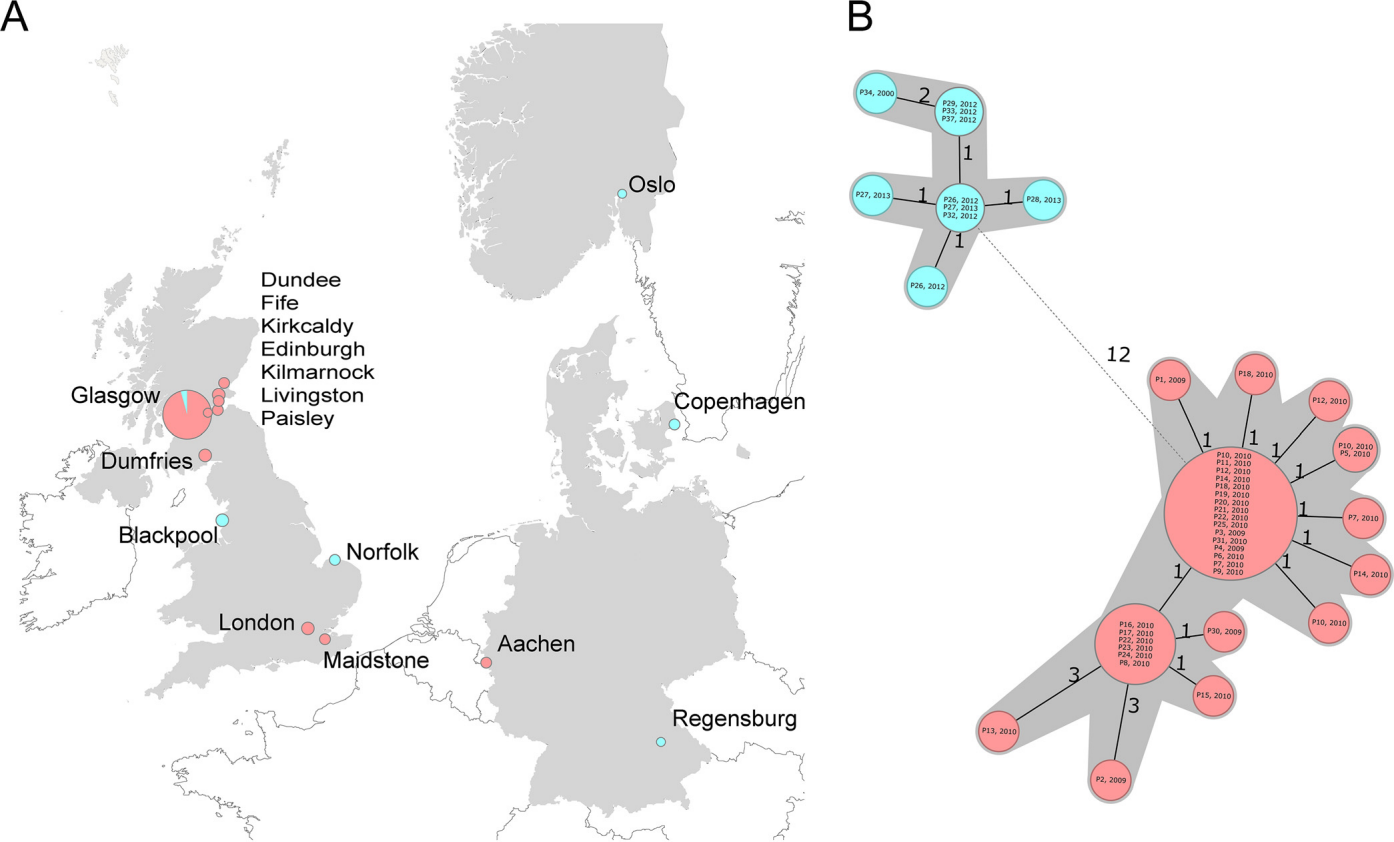
Geographical origin distribution (A) and minimum-spanning tree (B) illustrating 57 B. anthracis strains from human cases (heroin users) with injectional anthrax in four different European countries. Each node represents a unique cgMLST allele profile. The sizes of the nodes represent the number of isolates. Colored nodes represent the different clusters identified based on whole-genome SNPs and cgMLST. Numbers on connecting lines refer to the numbers of different alleles. cgMLST profiles with less than five different alleles to the central genotype are shaded.

To evaluate this assumption, we challenged the newly developed cgMLST and the proposed cutoff for clustering. We investigated 57 published B. anthracis strains from heroin users in four different European countries (Data Set 3 in Table S1). The strains were isolated from human cases with injectional anthrax, with in some cases several isolates being recovered from a single patient (26). As shown in Fig. 4, the cgMLST splits these strains into two groups, with 12 allele differences (corresponding to an average of 13.5 SNPs). A maximum of two allele differences was observed for the strains derived from a single patient. Additionally, with the exception of two strains, five alleles represented the maximum allele variation within each group. Each group had averages of 3.1 and 3.88 SNPs for groups I and II, respectively. These results are concordant with the published SNP analysis in which Keim and colleagues (26) identified two tight clusters, pointing toward two disease events, probably associated with two incidents of drug contaminations.

### Availability of B. anthracis cgMLST profiles.

In order to facilitate cooperation in the control of B. anthracis infections at a global level, we aimed for a unified web-accessible nomenclature. Therefore, the developed cgMLST scheme was made publicly available at https://pubmlst.org/ (19). New strains can be typed and compared in real time with a global collection of currently 684 B. anthracis genomes (November 2020) available in the database. In addition, the database includes a hierarchy of cluster definitions for the B. anthracis cgMLST profiles at thresholds of 200, 100, 50, 25, 10, and 5 different alleles, using the single-linkage method. This is in order to explore potential local and regional transmission chains of B. anthracis strains in future investigations. Along with cgMLST targets, 1,263 accessory genes are hosted at PubMLST that can be combined with the cgMLST profiles to perform a whole-genome-based MLST (wgMLST) to further improve interstrain resolution if required.

Besides PubMLST, we applied the freely available tool chewBBACA (18) to the data set investigated in this study. The typing results and the formatted database are available via figshare (https://doi.org/10.6084/m9.figshare.13220735). All cgMLST profiles defined using SeqSphere for the 584 B. anthracis strains in the evaluation data set have been correctly identified using chewBBACA. However, chewBBACA split some cgMLST profiles into several profiles, leading to a slightly larger number of profiles being described. A total of 499 cgMLST profiles were defined using chewBBACA, compared to 473 profiles identified using SeqSphere (missing alleles were ignored in the comparison), resulting in a Simpson’s index of diversity of 0.999 (95% CI, 0.998 to 0.999). Using cgMLST results from chewBBACA as the primary typing method, the adjusted Wallace value was 0.995 (95% CI, 0.990 to 1.000), while using SeqSphere as the primary typing method, the adjusted Wallace value was 0.678 (95% CI, 0.567 to 0.790).

## DISCUSSION

Whole-genome sequencing has been introduced in clinical diagnostics as a powerful tool. The so-called “next-generation sequencing” (NGS) technologies generate sequence data for bacterial pathogens at high throughput, at affordable costs, and with high speed. Due to the advantages it offers, the use of NGS in clinical laboratories has become essential for outbreak investigations and surveillance. However, challenges associated with NGS include bioinformatics analysis and standardized analytic workflows and nomenclature for pathogens and clades. In the pregenomic era, MLST was regarded as the “gold standard” for typing of many pathogens thanks to the unified allele nomenclature and centralized gene allele library that can be accessed via public web services: e.g., https://pubmlst.org/. However, the method suffers poor resolution for B. anthracis, which is a highly monomorphic and evolutionarily stable pathogen. The high virulence potential of this pathogen, its worldwide and unknown dispersal of spore-contaminated fields (“champs maudit”) in the environment but also its potential use as an agent for bioweapons motivate in-depth genotyping for routine surveillance and outbreak investigation. As such, whole-genome SNP analysis has been applied by many laboratories for detailed subtyping of B. anthracis (5, 15). However, the use of custom software and in-house workflows for SNP analysis affects the reproducibility and standardization of SNP typing approaches. Our study presents the first application of a whole-genome allele typing system to B. anthracis. Coupled with a centralized publicly accessible database for allelic profiles, cgMLST can provide a platform to augment SNP analysis to allow faster communication at international scales for tackling outbreaks as well as to enhance strain genotyping results for longitudinal and cross-sectional surveillance activities. The supported establishment of free software tools (39) together with easy-to-use online systems for cgMLST (19) may facilitate the utilization of WGS by laboratories with little bioinformatics knowledge.

The application of the B. anthracis cgMLST scheme involved the analysis of an extensive collection of B. anthracis genomes with very different geographical origins to determine the species population structure. The explicit definition of B. anthracis clades and subclades using cgMLST was characterized by a high degree of congruence to the well-established and widely used canonical SNP nomenclature. The results were also very consistent with the methods for SNP typing of the whole genome (20), despite the fact that MLST reduces genetic variations in genes to a single allelic difference, and distance-based phylogenetic analysis of MLST does not account for the full phylogenetic information of sequence data. However, in B. anthracis, SNP variations in cgMLST genes were minimal (median, 2 SNPs; mean, 2.7 ± 2.9 SNPs), corresponding to a low number of alleles (median, 3 alleles; mean, 3.6 ± 2.4 alleles) in all genomes studied. Nevertheless, shifts in the phylogenetic positions of a few groups within the TEA subclade (A.Br.008) were observed in the cgMLST-based phylogenetic tree. This can be attributed to the abundance of simultaneous descendant lineages and the frequent occurrence of extremely short phylogenetic branches in the TEA subclade (7, 20), for which the character-based phylogenetic methods using whole-genome SNP data can be more accurate.

The current implementation of allele typing involves the use of assembled genomes as input for gene detection and allele assignment (16, 17), a process that does not inherently employ FASTQ reads to identify spurious variants; hence, the quality of assembled data is crucial for correct allele typing (16, 40–42). The results of cgMLST can be detrimentally compromised by (i) sequencing errors that impair the fidelity of genome assembly, such as erroneous bases, indels, or frameshifts, and (ii) fragmentation of genome assembly, which results in missing or incomplete genes. As such, cgMLST typing tools assign allele numbers only to complete genes without ambiguities or internal stop codons (17, 19). The numbers of loci detected and alleles typed per strain are reflective of the quality of the assembled genomes, which can also be monitored by a number of metrics, such as *N*_50_, total assembly size, and percentage of genome alignment to the reference (28). Similar to highly clonal species (43), we arbitrarily set that at least 96% of cgMLST loci are found and typed as a prerequisite for core genome sequence type (cgST) assignment and clustering. Although it can be challenging to assess the correctness of the assembled genomes, studies have shown that the greater sequencing depth for Illumina data can ameliorate the effect of using different assemblers, optimize genomes’ contiguity, and help improve the accuracy and reproducibility of cgMLST calls (17, 40, 44).

In this study, we set a cutoff for strain typing based on the *N*_50_ values as an indication parameter for improved genome contiguity. However, 12 genomes of sufficient assembly quality were poorly typeable, with less than 90% of good cgMLST targets being detected. Further analysis revealed that these 12 genomes do not belong to the classical highly clonal B. anthracis lineage following Carroll and colleagues’ proposal (6), in which strains of classical B. anthracis lineage should have more than 99.9% ANI compared to the Ames reference genome. The 12 genomes have ANI values below 98% and were phylogenetically positioned basal to known A, B, and C clades of B. anthracis based on SNP analysis, while one strain (out of 12 strains) carried the anthrax plasmid genes of pXO2 (Table S1). These results on one hand indicate that the developed cgMLST system can reliably distinguish the classical B. anthracis lineage (clades A, B, and C) without the need for a prior estimation of ANI for the strains or phenotypic identification as shown with cgMLST (e.g., for *Yersinia* spp. [45]). On the other hand, the fact that anthrax plasmids can be easily transferred to other B. cereus strains may impede proper strain characterization by classical methods. The burden of plasmid spread among related strains of B. cereus necessitates proper identification of the highly clonal B. anthracis lineage, which is also important for epidemiological and forensic applications and can be achieved using this cgMLST scheme.

Besides cgMLST, options for bacterial strain typing include among others whole-genome MLST (core and accessory genomes) and reference-based SNP approaches, which can provide slightly higher resolution than cgMLST (16). However, the discrimination level obtained by cgMLST was sufficient for epidemiological investigations of German and Italian anthrax outbreaks as well as injectional anthrax cases in different European countries. A total of 3,803 coding DNA sequences (CDSs [70%]) of 5,357 CDSs present in the chromosome of the reference Ames Ancestor strain proved to be helpful for high-resolution typing. This is comparable to other highly clonal bacteria such as Brucella melitensis and Francisella tularensis, where 81 and 65% of the genes with coding sequences are used, respectively (46, 47).

In conclusion, we present a cgMLST scheme that provides a high-resolution strain genotyping for the highly monomorphic bacterium B. anthracis. This scheme reliably identifies B. anthracis strains down to the strain level and can be used in parallel with SNP typing methods to facilitate rapid and harmonized interlaboratory comparisons, essential for global surveillance and outbreak analysis.
