# Supplementary material for: A Whole-Genome-Based Gene-by-Gene Typing System for Standardized High-Resolution Strain Typing of Bacillus anthracis
Source: J Clin Microbiol. 2021 Jun 18;59(7):e02889-20. doi: 10.1128/JCM.02889-20 (PMC8218748; doi:10.1128/JCM.02889-20)
Supplement: Supplemental file 1 — . Download JCM.02889-20-s0001.pdf, PDF file, 1.39 MB [file jcm.02889-20-s0001.pdf]

Supplemental Material (Supplemental Figures S1 – S2 and legends of Supplemental Tables S1 - S4)

A whole genome-based gene-by-gene typing system for standardised high resolution strain typing of *Bacillus anthracis*

Mostafa Y. Abdel-Glil[1,2#], Alexandra Chiaverini[3], Giuliano Garofolo[3], Antonio Fasanella[4], Antonio Parisi[4], Dag Harmsen[5], Keith A Jolley[6], Mandy C. Elschner[1], Herbert Tomaso[1], Jörg Linde[1], Domenico Galante[4]

---

[1]Institute for Bacterial Infections and Zoonoses, Friedrich-Loeffler-Institut, Jena, Germany

[2]Department of Pathology, Faculty of Veterinary Medicine, Zagazig University, Sharkia Province, Egypt, Egypt

[3]Istituto Zooprofilattico Sperimentale dell'Abruzzo e del Molise "G. Caporale", Teramo, Italy

[4]Anthrax Reference Institute of Italy, Istituto Zooprofilattico Sperimentale della Puglia e Basilicata, Foggia, Italy

[5]Department of Periodontology and Operative Dentistry, University Hospital Muenster, Muenster, Germany

[6]Department of Zoology, University of Oxford, Oxford, United Kingdom.

Supplemental Figures S1 – S2

Supplemental Figure S1: Representativeness of *B. anthracis* strains selected and used for the calculation of the species core genome. Maximum likelihood phylogenetic tree constructed using 9,506 whole-genome SNPs based on a data set of 172 *B. anthracis* genomes that were downloaded from the RefSeq database. The corresponding genetic groups of the strains are shown in the inner column. Further annotation of the genomes in regard to genetic diversity was indicated in the outer columns numbered 1, 2 and 3 based on hierBAPS groups according to Bruce et al., 2019, updated canonical SNPs typing from Sahl et al., 2016 and traditional canonical SNPs grouping from Van Ert et al., 2007, respectively. Red dots at the tip of the branches indicate selected genomes for the calculation of the species core genome.

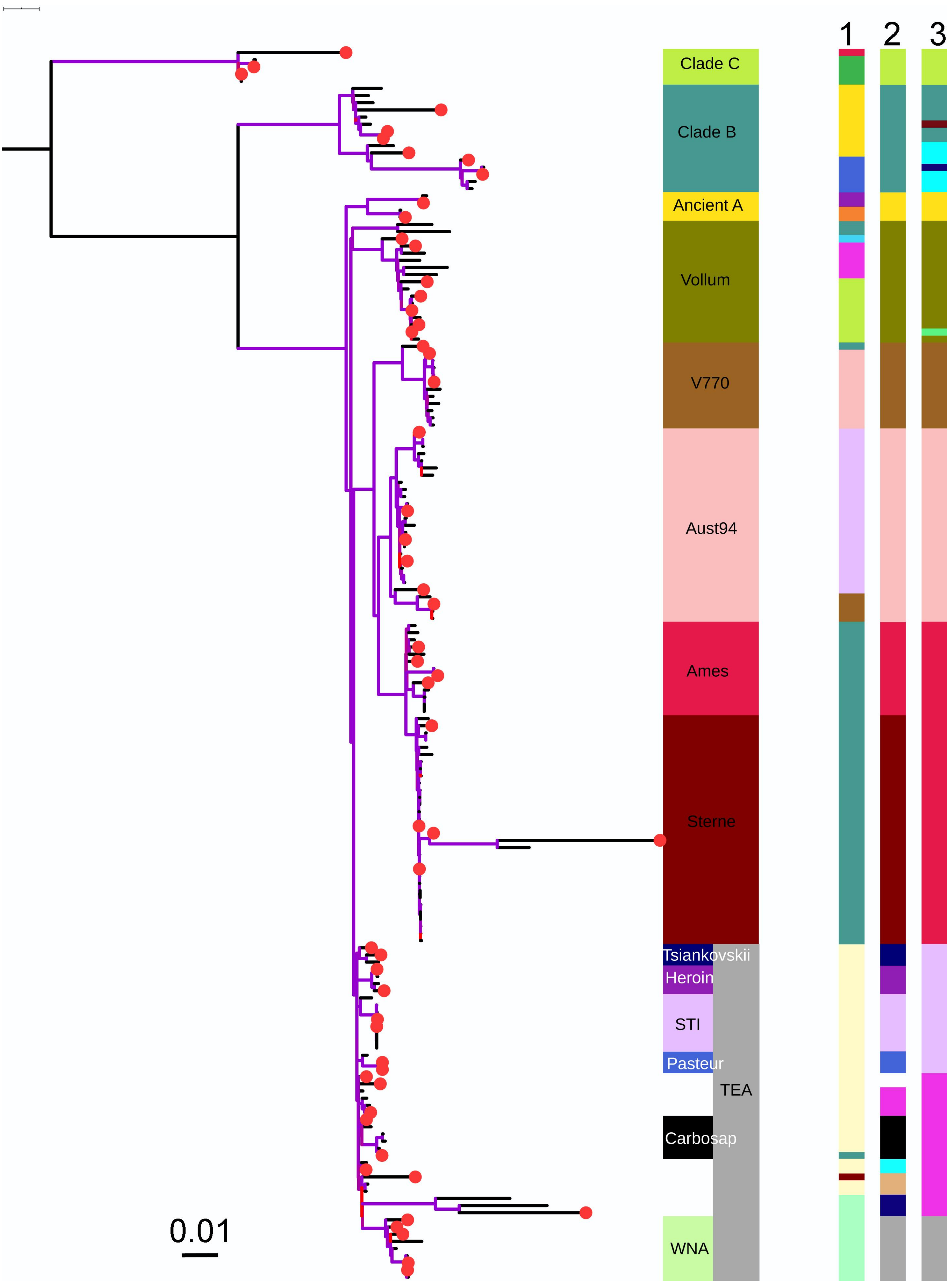
1
2
3
Clade C
Clade B
Ancient A
Vollum
V770
Aust94
Ames
Sterne
Tsiankovskii
Heroin
STI
Pasteur
TEA
Carbosap
WNA
0.01

Supplemental Figure S2: Topological concordance between the cgMLST neighbor joining tree (left) and the SNP-based maximum likelihood tree (right) is represented as tanglegram. Tree branches were coloured based on the major clades of *B. anthracis*, clade A: blue, clade B: red and clade C: green.

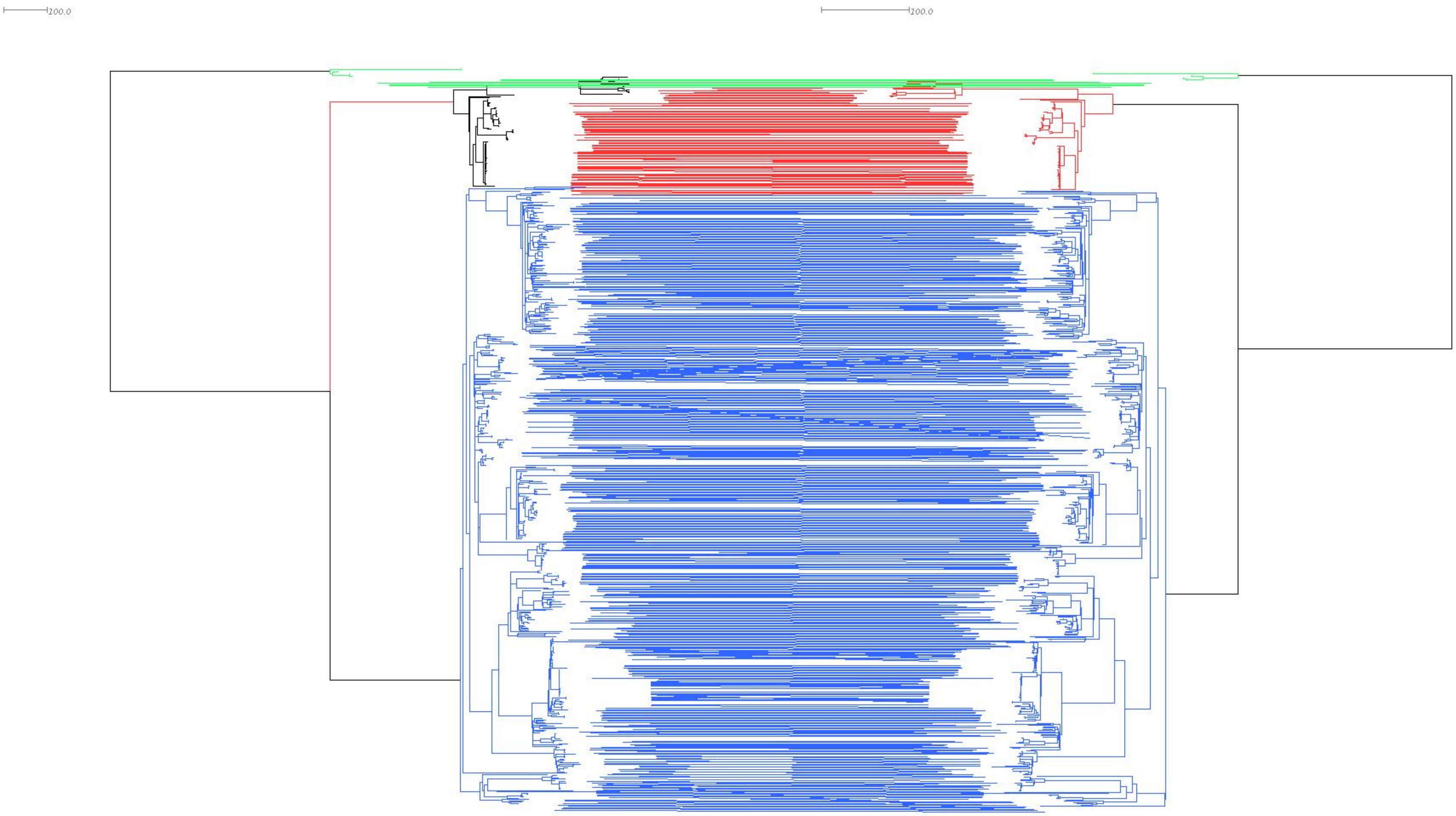
200.0
200.0

Legends of Supplemental Tables S1 - S4

Supplemental Table S1: Metadata information of strains and genomes of *B. antharcis* used in the setup and application of the core genome MLST system.

Supplemental Table S2: List and characteristics of the 3803 core genome MLST target genes used for *B. anthracis*.

Supplemental Table S3: Typeability percentages and variability of *B. anthracis* clades, average allele distance within and between each of the identified (sub)clades as well as the average allele distance of each (sub)clade to the closest sample in the other groups.

Supplemental Table S4: group-specific SNPs that are specifically present in each of the *B. anthracis* (sub)clade but simultaneously absent in all other genomes.
